# Epidemiology and Clinical Impact of Confirmed Respiratory Viral Infections in Solid Organ Transplant Recipients

**DOI:** 10.1111/tid.70128

**Published:** 2025-11-08

**Authors:** Manon L. M. Prins, Ernst D. van Dokkum, Aiko P. J. de Vries, Maarten E. Tushuizen, Danny van der Helm, Edwin M. Spithoven, Irene M. van der Meer, Eduard M. Scholten, Albert M. Vollaard, Saskia le Cessie, Leo G. Visser, Geert H. Groeneveld

**Affiliations:** ^1^ LUCID‐Subdepartment of Infectious Diseases Leiden University Medical Center Leiden the Netherlands; ^2^ Department of Internal Medicine Division Acute Internal Medicine Leiden University Medical Center Leiden the Netherlands; ^3^ Department of Public Health and Primary Care Leiden University Medical Center Leiden the Netherlands; ^4^ Health Campus Leiden University Medical Center The Hague the Netherlands; ^5^ Department of Internal Medicine Division of Nephrology and Leiden Transplant Center Leiden University Medical Center Leiden the Netherlands; ^6^ Department of Gastroenterology and Hepatology Leiden University Medical Center Leiden The Netherlands; ^7^ Department of Internal Medicine Amphia Hospital Breda the Netherlands; ^8^ Department of Internal Medicine Rijnstate Hospital Arnhem the Netherlands; ^9^ Department of Nephrology Haga Teaching Hospital The Hague the Netherlands; ^10^ Department of Nephrology Haaglanden Medical Center The Hague the Netherlands; ^11^ Centre for Infectious Disease Control National Institute for Public Health and the Environment Bilthoven the Netherlands; ^12^ Department of Clinical Epidemiology Leiden University Medical Center Leiden the Netherlands

**Keywords:** co‐infections, hospital admission, mono‐infections, morbidity, respiratory viral infections, solid organ transplant recipients

## Abstract

**Background:**

Respiratory viral infections (RVIs) can have distinct clinical presentations and outcomes in non‐lung solid organ transplant (SOT) recipients compared to non‐transplant and lung transplant patients. Understanding their impact is crucial for improving patient care and outcomes.

**Methods:**

This multicenter retrospective study analyzed adult non‐lung SOT recipients with PCR‐confirmed symptomatic RVIs from eight Dutch hospitals (January 2013–July 2024) to characterize clinical characteristics and outcomes of mono‐ and co‐infections and identify risk factors for intensive care admission or 30‐day mortality.

**Results:**

In total, 603 RVIs were identified in 460 recipients (kidney: 501; liver: 75; pancreas/islet of Langerhans: 4; combined: 23). The most common viruses were SARS‐CoV‐2 (36%), influenza A/B (29%), rhinovirus (14%), and RSV (7%). Influenza cases showed higher rates of fever (72%), common cold symptoms (37%), and myalgia (29%) than other viruses. Hospitalization occurred in 68% (384/565). Factors independently associated with intensive care admission or 30‐day mortality included higher CURB‐65 score (OR 1.91; 95% CI 1.36–2.70; *p* < 0.01), radiologic infiltrates (OR 3.04; 95% CI 1.60–5.80; *p* < 0.01), and SARS‐CoV‐2 infection (OR 1.67; 95% CI 1.05–2.67; *p* = 0.03). In contrast, influenza infection was associated with a lower risk (OR 0.21; 95% CI 0.07–0.62; *p* < 0.01). Co‐infections were not linked to worse outcomes compared to mono‐infections.

**Conclusion:**

Overall, RVIs in non‐lung SOT recipients were associated with high hospitalization and mortality rates. SARS‐CoV‐2 posed the highest risk for complications, while influenza was associated with a lower risk of severe outcomes. No association was found between co‐infection and poor outcomes.

## Graphical abstract

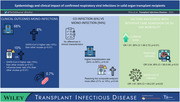

**Abbreviations**
ATG: anti‐thymocyte globulin
AUC: area under the curve
CVD: cardiovascular disease
DM: diabetes mellitus
HAdV: human adenovirus
HBOV: human bocavirus
HCoV: human coronavirus
HMPV: human metapneumovirus
HPIV: human parainfluenza
HRV: human rhinovirus
ICU: intensive care unit
IQR: interquartile range
LUMC: Leiden University Medical Center
OR: odds ratio
RSV: respiratory syncytial virus
RVI: respiratory viral infection
SD: standard deviation
SOT: solid organ transplant

## 1 Introduction

Community‐acquired respiratory viral infections (RVIs) are caused by viruses like influenza, human parainfluenza (HPIV), human rhinovirus (HRV), human coronaviruses (HCoV), human metapneumovirus (HMPV), and respiratory syncytial virus (RSV). While these infections are typically mild in healthy individuals, solid organ transplantat (SOT) recipients may experience a typical, delayed or exacerbated presentations due to their compromised immune system [1, 2, 3]. In addition, disruption in critical elements of both the innate and adaptive immune response may result in uncontrolled viral replication and consequent progression of the infection to the lower respiratory tract [2, 4, 5, 6], thereby leading to substantial morbidity and mortality [7, 8, 9].

Most research on RVIs in SOT recipients focuses on lung transplant recipients, with limited data on non‐lung SOT recipients, who suffer from systemic compromised immune response but do not have the transplant as the site of infection. Additionally, while common viruses like SARS‐CoV‐2, influenza, and RSV are well‐studied, less common viruses like HMPV, HPIV, and human bocavirus (HBOV) are not. Understanding the disease burden of these pathogens is essential, especially with the development of new vaccines, including those for RSV, HMPV, and HPIV [10]. Therefore, we performed a multicenter retrospective study to examine the impact of several RVIs in non‐lung SOT recipients.

## 2 Methods

### 2.1 Study Design

This multicenter retrospective observational study includes data from the Leiden University Medical Center (LUMC) and seven affiliated non‐academic hospitals. The LUMC, one of seven transplant centers in the Netherlands, annually performs 170–200 transplants, mostly kidney transplants. Inclusion criteria were adult (≥ 18 years) SOT recipients (kidney, liver, pancreas, islet cells of Langerhans, or combinations) with microbiologically confirmed RVIs via PCR (nasopharyngeal or throat swab, sputum, or bronchoalveolar lavage) from January 1, 2013 until July 1, 2024. Excluded were thoracic SOTs (these are not performed in the LUMC) and hospital‐acquired RVIs (symptoms starting ≥ 48 h post‐admission). Patients must have been symptomatic (fever and/or symptoms of an RVI) and tested for viruses beyond just SARS‐CoV‐2, as PCR for SARS‐CoV‐2 was used as a screening method during the COVID‐19 pandemic. Multiple RVI episodes per patient were allowed, provided that there was at least a 3‐month gap between two infections with the same virus. The selection of pathogens tested by PCR was determined by the treating physician and hospital.

Clinical characteristics were collected from medical records, including demographics, symptoms, comorbidities, CURB‐65 score, laboratory parameters (lymphocyte count and urea), chest x‐ray infiltrates at admission, medication use, and outcomes. Comorbidity was categorized into cardiovascular disease (CVD), chronic pulmonary disease and diabetes mellitus (DM). Immunosuppression levels were based on the number and type of medications, with triple therapy and/or lymphocyte‐depleting agents (anti‐thymocyte globulin [ATG] or alemtuzumab) in the preceding 6 months indicating high immunosuppression. Viral co‐infection was defined as multiple pathogens testing positive on PCR at admission. Rejection was reported if it was proven through a biopsy or if patients were treated with rejection treatment.

### 2.2 Laboratory Methods

Respiratory samples were processed using a range of molecular diagnostic platforms across participating hospitals. Most hospitals employed GeneXert (Cepheid) for rapid, cartridge‐based real‐time (qPCR) testing. Additional high‐throughput platforms included Alinity m (Abbott), Cobas 6800 (Roche), and Panther Fusion (Hologic), all utilizing qPCR for respiratory pathogen detection. Some centres also used Fast Track diagnostics assays (Siemens) and locally developed laboratory assay (LDTs). In one hospital, additional testing was performed using RespiFinder Smart/ 2Smart (Pathofinder) and ID Now (Abbott), enabling rapid point‐of‐care molecular diagnostics. Across all platforms, real‐time PCR was the predominant method for the detection of respiratory viruses.

### 2.3 Objectives

Primary outcomes included the number of confirmed RVIs in SOT recipients and their clinical characteristics, including clinical presentation. Secondary outcomes included clinical outcomes like hospitalization, length of stay, intensive care unit (ICU) admission, mechanical ventilation, 30‐day mortality, organ transplant rejection < 30 days after hospital admission, and a composite endpoint of 30‐day mortality or ICU admission, with the identification of factors associated with this composite endpoint. Outcomes were compared across different viral types and between mono‐infections and co‐infections.

### 2.4 Statistical Analysis

Descriptive statistics were used to report clinical characteristics, with continuous variables presented as means and standard deviations (SDs) or medians and interquartile range (IQR), depending on distribution. Categorical variables were reported as numbers and percentages. Respiratory viruses identified less than 20 times over a 10‐year period were grouped into a “residual” category, with non‐SARS‐CoV‐2 coronaviruses and parainfluenza viruses also aggregated. Patients with positive tests for multiple respiratory viruses were excluded from analyses of infection characteristics and clinical outcomes, as it was unclear which virus contributed to the disease burden and symptoms. They were classified as a separate group (co‐infections). Clinical outcomes between different viral types were compared using ANOVA, Kruskal–Wallis or chi‐squared test, with post‐hoc analysis in case of significance (*p* < 0.05) and Bonferroni correction for multiple testing. If a post‐hoc analysis was performed, the Bonferroni‐corrected *p* value is reported in the text. Co‐infection versus mono‐infection outcomes were assessed using *t*‐tests, Mann–Whitney *U* or chi‐squared test, with significance set at *p* < 0.05.

We assessed the association of certain patient characteristics with the composite endpoint (30‐day mortality or ICU admission) through multivariable logistic regression analysis. Variables significantly associated (*p* value < 0.10) in univariate analysis, along with clinically relevant factors identified a priori (age, highly immunosuppressed status, use of proliferation inhibitors such as mycophenolate mofetil), were included in the model. Backward selection was used to eliminate non‐significant predictors. For this model, all patients were included, regardless of co‐infection status.

Calculations were performed using SPSS Statistics 25.0 for Windows. Figures were made using GraphPad Prism version 9.3.1 for Windows, San Diego, California.

### 2.5 Reporting and Ethics

The study was done in accordance with Good Clinical Practice Guidelines and approved by the Institutional Review Board of the LUMC (nWMODIV2_2022034), which waived the need for informed consent. Additionally, the institutional review boards of the individual sites also approved the study. The study was reported following the guidelines for observational studies.

## 3 Results

During the study period, 643 viruses were detected in 603 positive PCR tests among 460 individual patients (kidney, *n* = 501; liver, *n* = 75; pancreas/ islet of Langerhans, *n* = 4; combined transplant, *n* = 23) (Figure S1). Of these, 143 (24%) had a previous positive PCR test and were therefore included more than once. Co‐infections occurred in 38 episodes (6%), including 36 double and 2 triple infections (Table S1), with the remaining 565 being mono‐infections. In 19 co‐infections (50%), influenza A and/or B was one of the viruses, while in 16 co‐infections (42%) SARS‐CoV‐2 was involved. There were six cases (16%) of influenza/ SARS‐CoV‐2 infection and six cases (16%) of SARS‐CoV‐2/RSV infection. No differences were observed between mono‐ and co‐infected patients (Table 1).

**TABLE 1:** Patient characteristics.

	Mono‐infections (*n* = 565)	Co‐infections (*n* = 38)	*p*
Age, mean (SD)	59 (14)	61 (11)	0.32
Male sex, *n* (%)	331 (59)	28 (74)	0.07
BMI, mean (SD)	25.6 (4.9)	25.0 (4.0)	0.48
Time from transplantation in years, median (IQR)	5 (2‐11)	4 (2‐13)	0.66
Co‐morbidities, *n* (%)	503 (89)	35 (92)	0.55
Pre‐existent CVD disease	469 (93)	31(89)	0.82
Pre‐existent lung disease	155 (31)	13(37)	0.37
Pre‐existent DM	230 (46)	20(57)	0.15
Type transplantation, *n* (%)			0.54
Kidney	470 (83)	31 (82)	
Liver	71 (13)	4 (11)	
Combined^a^	20 (4)	3 (8)	
Pancreas or islets of Langerhans^b^	4 (1)	0 (0)	
Induction immunosuppression, *n* (%)^c^			0.40
IL‐2 inhibitor	357 (89)	22 (79)	
ATG	1 (0.2)	0 (0)	
Alemtuzumab	38 (10)	5 (18)	
Other	6 (2)	1 (4)	
No. of immunosuppressive agents			0.35
1	54 (10)	2 (5)	
2	277 (49)	23 (61)	
3	234 (41)	13 (3)	
Maintenance immunosuppressive agents			
Corticosteroids	495 (88)	36 (95)	0.19
Calcineurin inhibitors	425 (75)	25 (66)	0.20
Proliferation inhibitors	346 (61)	20 (53)	0.29
MTOR inhibitors	38 (7)	5 (13)	0.14
Previous rejection therapy			
<6 months ago	12 (2)	0 (0)	0.45
Once	104 (18)	5 (13)	
Never	449 (80)	33 (87)	
Type of rejection therapy^c^			
Solumedrol	96 (17)	4 (11)	0.29
Alemtuzumab	28 (5)	0 (0)	0.16
ATG	20 (4)	2 (5)	0.59
Other^d^	29 (5)	1 (3)	0.49
			
Time between rejection therapy and PCR in years, median (IQR)	4 (1‐13.8)	17 (1.5‐21.5)	0.40
Highly immunosuppressed^e^	235 (42)	13 (34.)	0.37
Clinical presentation, *n* (%)			
Fever	326 (58)	16 (42)	0.16
Coughing	397 (70)	31 (82)	0.33
Sore throat	78 (14)	4 (11)	0.82
Dyspnoea	221 (39)	17 (45)	0.77
Common cold	152 (27)	9 (24)	0.88
Myalgia	101 (18)	6 (16)	0.92
Headache	98 (17)	3 (8)	0.31
General malaise	164 (29)	11 (29)	0.99
Other^f^	55 (10)	4 (11)	0.78
Time between first symptoms and PCR in days, median (IQR)	3 (1‐7)	4 (2‐8.5)	0.30
Time between first symptoms and admission in hospital in days, mean (SD)	4.8 (7.7)	6.9 (11.9)	0.18
CURB‐65 score, mean (SD)	1.5 (0.9)	1.4 (0.9)	0.80
Radiology, *n* (%)	421 (75)	31 (82)	0.33
Infiltrate	130 (31)	9 (29)	0.83
Treated with oseltamivir, *n* (%)	110/166 (66)	12/19 (32)	0.88
Antibiotics prior to admission, *n* (%)	171 (30)	9 (24)	0.39
Antibiotics after admission, *n* (%)	334 (59)	26 (63)	0.26

*Note*: Univariate analysis was performed for the baseline characteristics using either the Mann–Whitney *U* test or chi‐squared test.

Abbreviations: ATG, anti‐thymocyte globulin; BMI, body mass index; CVD, cardiovascular disease; DM, diabetes mellitus; HCoV, human coronavirus; HMPV, human metapneumovirus; HPIV, human parainfluenza virus; HRV, human rhinovirus; I, interleukin‐2; influenza, influenza virus; MTOR, mammalian target of rapamycin; RSV, respiratory syncytial virus; SARS‐CoV‐2, severe acute respiratory syndrome coronavirus‐2.

^a^ Combined transplantation include kidney–pancreas (*n* = 12) and kidney–liver (*n *= 11).

^b^ Pancreas transplantation: *n *= 2; Islets of Langerhans: *n* = 2.

^c^ Valid percentages, as there are some missing data.

^d^ Other types of rejection therapy included muromonab‐CD3 (OKT3), plasmapheresis, IVIG, rituximab, addition of a third agent, switch to tacrolimus.

^e^ Included patients who used lymphocyte depleting agents less than 6 months ago (*n *= 5) and/or patients who used three immunosuppressive agents (*n* = 247). Four of the five patients who used lymphocyte depleting agents used three immunosuppressive agents as well.

^f^ Other symptoms include tiredness, chest pain, cold chills, ear pain, delirium, and need of oxygen.

When comparing patient characteristics between different respiratory viruses (Table S2), a difference remained only after post‐hoc testing regarding age. Patients with influenza were younger than those with SARS‐CoV‐2, RSV, and HMPV, with *p* values of < 0.01, < 0.01, and 0.04, respectively.

Between 2017 and 2020, more extensive testing (> 10 viruses per PCR) was conducted; before 2017 and after 2020, PCR tests more frequently tested for 0–5 pathogens (influenza A and B, RSV, and, after 2019, SARS‐CoV‐2) (Figure S2; Tables S3 and S4). In the academic hospital, more viruses were tested per PCR than in the non‐academic hospitals (median [IQR] 15 [4–15] vs. 4 [4–15], respectively, *p* < 0.01).

### 3.1 Distribution of RVIs

SARS‐CoV‐2 (36%), influenza A and/or B (29%), HRV (14%), and RSV (7%) were the most common causes of RVIs. Less frequently detected viruses, including human adenovirus (HAdV), HBoV, and enterovirus, were grouped into the “residual” group. While HPIV, HRV, HBoV, enterovirus, and SARS‐CoV‐2 were present year‐round, influenza and RSV predominantly circulated during the winter months (Figure S3A). HCoV were detected in autumn, winter, and spring, while HAdV primarily appeared in winter and spring.

In the influenza group, 74% (137/185) had influenza A and 26% (48/185) influenza B. Among 24 HPIV infections, the majority were caused by parainfluenza 3. In 2022, there was an increase in SARS‐CoV‐2 cases, as well as in infections caused by HMPV, influenza, and RSV (Figure S3B).

### 3.2 Clinical Characteristics

**FIGURE 1:**
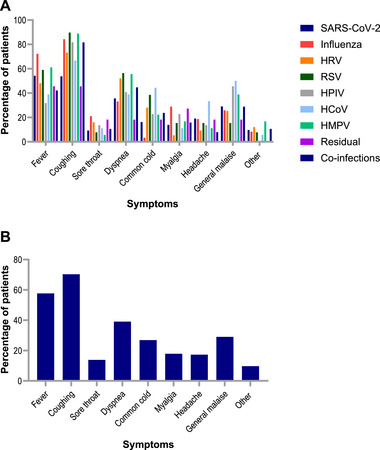
Symptoms during clinical presentation (%). Symptoms at the time of presentation in patients with a mono‐infection (*n *= 565) and with a co‐infection (*n* = 38; shown separately, see blue bars), stratified by virus. The “residual” group consists of the adenovirus, bocavirus, and the enterovirus. HCoV, human coronavirus; HMPV, human metapneumovirus; HPIV, human parainfluenza virus; HRV, rhinovirus; RSV, respiratory syncytial virus; SARS‐CoV‐2, severe acute respiratory syndrome coronavirus‐2.

All respiratory viruses generally cause similar symptoms (Table S2 and Figure 1). Regarding clinical characteristics, no differences were observed between mono‐ and co‐infected patients (Table 1). In mono‐infected patients, coughing was most common in influenza cases (140/166; 84%) and less in SARS‐CoV‐2 (116/216; 54%) (*p* < 0.01). Fever was more frequently reported in influenza infections (120/166; 72%) compared to other RVIs (*p* < 0.01). Influenza patients also reported more common cold symptoms (62/166; 37%; *p* < 0.01), while those with SARS‐CoV‐2 reported less common cold symptoms (35/216; 16%; *p* < 0.01). Myalgia was also more common in influenza patients (48/166; 29%; *p* < 0.01), and rare in HRV patients (4/75; 5%; *p* < 0.01).

The mean (SD) CURB‐65 score of all patients with an RVI was 1.5 (0.9). Chest x‐ray was performed in 75% (421 / 565) of mono‐infections, though it was less frequently done for SARS‐CoV‐2 (136/216; 63%) compared to all viruses (*p* < 0.01). Radiologic infiltrates were observed in 130/421 (31%).

Of the 185 influenza positive and 232 positive SARS‐CoV‐2 patients (including both co‐ and mono‐infections), the vaccination status was known for 177 and 203 patients, respectively. Of these, 75 (42.4%) and 177 (87.2%) had been vaccinated.

### 3.3 Treatment of RVIs

Prior to admission, antibiotics were prescribed to 30% (171/565) of patients with mono‐infection RVIs and 24% (9/38) with co‐infections (Table 1 and Table S2). Mono‐infected influenza patients were less likely to receive antibiotics (35/166; 21%; *p* < 0.01), while mono‐infected HRV patients received antibiotics more often (36/75; 48%; *p *< 0.01). After admission, 59% (334/565) of mono‐infection patients and 63% (26/38) of co‐infection patients received antibiotics. Among the 360 patients who received antibiotics after presentation, 113 (37%) had an infiltrate on chest x‐ray. Mono‐infected HRV patients were more likely to receive antibiotics (59/75; 78%; *p* < 0.01), whereas mono‐infected SARS‐CoV‐2 patients were less frequently treated with antibiotics (109/216; 51%; *p* < 0.01).

Oseltamivir for influenza‐positive patients was administered to 122 out of 185 patients (66%) after lab confirmation. In total, 71 of the 232 SARS‐CoV‐2 positive patients (31%) were treated with at least one class of drugs, of which 42 (18%), 22 (10%), and 6 (3%) patients were treated with one, two, and three classes of drugs, respectively (total of 105 prescriptions). Of these prescriptions, corticosteroids were administered 49 times (47%), monoclonal antibodies 23 times (22%), an IL‐6 inhibitor 16 times (15%), and an antiviral 13 times (12%) (Figure S4).

### 3.4 Clinical Outcomes

Thirty‐day mortality in RVI patients was higher for those with a CURB‐65 score of 2–4 compared to those with a score of zero or one, even after excluding patients with an infiltrate on chest x‐ray (Tables S5 and S6).

In univariable models, the hospitalization and mortality rate for patients with mono‐infection RVI was 68% (384/565) and 6% (32/565), respectively (Table 2). Post‐hoc analysis revealed that SARS‐CoV‐2 had a higher mortality rate (22/216; 10.2%) compared to other RVIs (*p* < 0.01). Among mono‐infections, 10% (58/565) reached the composite endpoint, with SARS‐CoV‐2 having a higher rate (33/216; 15%) and influenza a lower rate (6/166; 4%; *p* < 0.01). Four patients (0.7%) developed graft rejection within 30 days, two from influenza, and two from HRV.

**TABLE 2:** Outcomes associated with RVI in patients with a mono‐infection.

Outcome variable	Total (*n* = 565)	SARS‐CoV‐2 (*n *= 216)	Influenza (*n *= 166)	HRV (*n *= 75)	RSV (*n *= 39)	HPIV (*n *= 22)	HCoV (*n *= 18)	HMPV (*n *= 18)	Residual^a^ (*n *= 11)	*p*
Hospital admission	384 (68.0)	154 (71.3)	102 (61.4)	49 (65.3)	28 (71.8)	15 (68.2)	14 (77.8)	13 (72.2)	9 (81.8)	0.45
Length of stay in days, median (IQR)	4 (2–8)	4 (2–12)	3 (2–5)	5 (2–12)	5 (2–8)	7 (3–10)	5 (3–8)	7 (4–8)	7 (2–12)	0.03
Admission to ICU	46 (8.1)	24 (11.1)	6 (3.6)	8 (10.7)	2 (5.1)	0 (0)	1 (5.6)	2 (11.1)	3 (27.3)	0.03
Mechanical ventilation	39 (6.9)	23 (10.6)	4 (2.4)	6 (8)	2 (5.1)	0 (0)	1 (5.6)	2 (11.1)	1 (39.1)	0.09
30‐Day mortality	32 (5.7)	22 (10.2)	3 (1.8)	3 (4.0)	1 (2.6)	0 (0)	1 (5.6)	0 (0)	2 (18.2)	0.006
Composite endpoint	58 (10.3)	33 (15.3)	6 (3.6)	10 (13.3)	2 (5.1)	0 (0)	2 (11.1)	2 (11.1)	3 (27.3)	0.003
30‐Day rejection	4 (0.7)	0 (0)	2 (1.2)	2 (2.7)	0 (0)	0 (0)	0 (0)	0 (0)	0 (0)	0.43

*Notes*: Counts and percentages are presented, unless otherwise stated. The composite endpoint consists of admission to ICU and 30‐day mortality.

Abbreviations: ICU, intensive care unit; IQR, interquartile range.

^a^ Residual category consists of the HBoV (human bocavirus), HAdV (human adenovirus), and the enterovirus.

Patients with co‐infections had a higher hospitalization rate (84% vs. 68%, odds ratio [OR] 2.51; 95% CI 1.03–6.12; *p* = 0.04) and reached the composite end point more often compared to those with mono‐infections (21.1% vs. 10.3%, OR 2.33; 95% CI 1.02–5.32; *p* = 0.04) (Table 3).

**TABLE 3:** Outcomes associated with mono‐infected and co‐infected RVIs.

Outcome variable	Mono‐infections (*n* = 565)	Co‐infections (*n *= 38)	OR (95% CI)	*p* ^a^
Hospital admission	384 (68.0)	32 (84.2)	2.51 (1.03‐6.12)	0.04
Length of stay in days, median (IQR)	4 (2–8)	7 (3–13)	—	0.22
Admission to ICU	46 (8.1)	5 (13.2)	1.71 (0.64–4.59)	0.28
Mechanical ventilation	39 (84.8)	4 (80.0)	1.59 (0.54–4.70)	0.34
30‐Day mortality	32 (5.7)	4 (10.5)	1.96 (0.65–5.85)	0.27
Composite endpoint	58 (10.3)	8 (21.1)	2.33 (1.02–5.32)	0.04
30‐Day rejection	4 (0.7)	0 (0)	0.99 (0.99–1.00)	1.00

*Note*: Counts and percentages are presented, unless otherwise stated. The composite endpoint consists of admission to ICU and 30‐day mortality.

Abbreviations: composite endpoint, 30‐day mortality and/or ICU admission; ICU, intensive care unit; IQR, interquartile range.

^a^ Mann–Whitney *U* test or chi‐squared test or Fisher's exact test (in the cells with a value of < 6).

Compared to influenza, SARS‐CoV‐2 infection was associated with an increased rate of hospitalization (OR 1.56; 95% CI 1.01–2.40; *p*  = 0.04) and ICU admission (OR 3.33; 95% CI 1.33–8.36; *p *< 0.01), mechanical ventilation (OR 4.83; 95% CI 1.64–14.24; *p* < 0.01), 30‐day mortality (OR 6.19; 95% CI 1.82–21.07; *p* < 0.01) and the composite endpoint (OR 4.81; 95% CI 1.96–11.77; *p* < 0.01) (Table S7).

RSV showed similar clinical outcomes to other RVIs like influenza and SARS‐CoV‐2, with no significant differences in outcomes (Tables S8 and S9). Outcomes for mono‐infected versus co‐infected patients with SARS‐CoV‐2 and influenza were similar (Tables S10 and S11), except for hospitalization rates in influenza patients (61% in mono‐infected patients vs. 95% in co‐infected patients; *p* < 0.01). Outcomes related to influenza disease were the same for vaccinated and unvaccinated patients (Table S12), in contrast to SARS‐CoV‐2 outcomes, where unvaccinated patients were more frequently admitted to the ICU and required ventilation than vaccinated patients (23% vs. 8%, respectively; *p*  =  0.02; Table S13).

SARS‐CoV‐2 positive patients treated with any medication were more frequently and for longer periods hospitalized and had a higher mortality rate than untreated SARS‐CoV‐2 patients (Table S14). Although SARS‐CoV‐2 outcomes seem more severe during the early stages of the pandemic, the differences are not statistically significant, except for ICU admission (Table S15).

### 3.5 Factors Associated With 30‐Day Mortality and / or ICU Admission

Risk factor analysis was conducted for the composite endpoint of 30‐day mortality or ICU admission. Univariate analysis identified several factors associated with the composite endpoint (Table 4). Multivariable logistic regression showed that after, backward selection, a higher CURB‐65 score (OR 1.91; 95% CI 1.36–2.70; *p* < 0.01), the presence of radiologic infiltrates (OR 3.04; 95% CI 1.60–5.80; *p* < 0.01) and SARS‐CoV‐2 infection (OR 1.67; 95% CI 1.05–2.67; *p*  =  0.03) were associated with ICU admission or 30‐day mortality. In contrast, the OR for influenza infection and ICU admission or 30‐day mortality was low (OR 0.21; 95% CI 0.07–0.62; *p* < 0.01).

**TABLE 4:** Factors associated with the composite endpoint due to RVI among all positive PCR tests (*n * =  603).

	OR (95% CI) (univariate analysis)	*p*	OR (95% CI) multivariate analysis	*p*
Age, mean (SD)	1.04 (1.0–1.1)	**<0.001**	—	—
Male sex	1.94 (1.1–3.4)	**0.02**	—	—
Type Tx				
Kidney	1	—	—	—
Liver	0.71 (0.29–1.70)	0.44		
Combined	1.71 (0.56–5.20)	0.35		
Pancreas and/or islets of Langerhans	2.70 (0.27–28.44)	0.39		
Years between transplantation and PCR, median (IQR)	1.01 (0.98–1.04)	0.50	—	—
Fever on presentation, *n* (%)	0.62 (0.37–1.04)	**0.07**	—	
Type of RVI (*n*)				
HCoV (25)	0.69 (0.16–3.00)	0.62	—	—
Influenza (185)	0.28 (0.13–0.60)	**0.001**	0.21 (0.07–0.62)	0.005
HMPV (21)	0.71 (0.54–0.93)	**0.013**	—	—
HPIV (24)	NA	NA	—	—
HRV (87)	0.75 (0.57–0.99)	**0.045**	—	—
SARS‐CoV‐2 (232)	1.34 (0.95–1.89)	**0.09**	1.67 (1.05–2.67)	0.03
RSV (45)	0.62 (0.30–1.26)	0.19	—	—
Residual^a^ (21)	1.96 (0.64–6.01)	0.24	—	—
Co‐infection	2.33 (1.02–5.32)	**0.045**	—	—
Highly immunosuppressed^b^	0.86 (0.51–1.45)	**0.57**	—	—
Maintenance immunosuppression, *n* (%)				
Calcineurin inhibitor	0.65 (0.37–1.12)	0.12	—	—
Proliferation inhibitor	1.24 (0.73–2.12)	**0.43**		
CURB‐65 score, mean (SD)	2.23 (1.64–3.03)	**<0.001**	1.91 (1.36–2.70)	<0.001
Radiologic infiltrate, *n* (%)	3.86 (2.22–6.70)	**<0.001**	3.04 (1.60–5.80)	<0.001
Previous rejection therapy (*n*)				
Never (109)	1.0	—	—	
<6 months ago (12)	1.65 (0.35–7.76)	0.52		
Once (482)	1.02 (0.53–1.99)	0.95		
Years between rejection and PCR, median (IQR)	1.01 (0.96–1.07)	0.61	—	—

*Note*: The composite endpoint consists of admission to ICU or 30‐day mortality. All variables that were significantly associated in the univariate analysis, along with clinically relevant variables, were included in the multivariate model (those in bold). In the multivariate model, only those variables that were significant predictors are shown. The non‐significant predictors were eliminated through backward selection.

Abbreviations: HAdV, human adenovirus; HBoV, human bocavirus; HCoV, human coronavirus; HMPV, human metapneumovirus; HPIV, human parainfluenza virus; HRV, human rhinovirus; ICU, intensive care unit; influenza, influenza virus; IQR, interquartile range; RSV, respiratory syncytial virus; SARS‐CoV‐2, severe acute respiratory syndrome coronavirus‐2.

^a^ Residual category consists of the HBoV (human bocavirus), HAdV (human adenovirus) and the enterovirus.

^b^ Included patients who used lymphocyte depleting agents less than 6 months ago (*n * =  5) and/or patients who used three immunosuppressive agents (*n*  =  247). Four of the five patients who used lymphocyte depleting agents used three immunosuppressive agents as well.

## 4 Discussion

In this large retrospective multicenter study of 460 non‐lung SOT recipients in the Netherlands, 603 microbiologically confirmed RVI episodes were identified between 2013 and 2024. SARS‐CoV‐2 and influenza were the most prevalent pathogens, with viral co‐infections occurring in 38 episodes (6%). Clinical presentation varied by pathogen, with influenza infections more frequently associated with coughing and fever, whereas SARS‐CoV‐2 infections were less commonly characterized by coughing. A higher CURB‐65 score, radiologic infiltrates and a SARS‐CoV‐2 infection were associated with poor outcome (ICU admission or 30‐day mortality), while influenza was associated with better outcomes.

RVIs remain a substantial health threat for SOT recipients, with an average mortality rate of 5.7% when considering all pathogens combined. Among these, influenza caused the least morbidity, with relatively low hospitalization (61%), ICU admission (4%) and 30‐day mortality (2%) rates, consistent with findings from Danish [8], Canadian [1] and Swiss [7] studies, the latter two also including lung SOT recipients. Unvaccinated status and delayed oseltamivir use are risk factors for poor outcomes in SOT recipients [1]. In our study, 42% of influenza positive patients were vaccinated, which aligns with the rates observed in recent years in the general population in the Netherlands [11] and is consistent with the results from a larger cohort of SOT recipients [12]. Influenza‐positive patients who were vaccinated showed outcomes similar to those of unvaccinated patients. Oseltamivir was given to two‐thirds of influenza‐positive cases in our cohort. However, the sample size was too small to draw conclusions regarding the efficacy of oseltamivir.

SARS‐CoV‐2 caused more severe disease than other RVIs, especially influenza, with higher ICU admission (11%), mechanical ventilation (11%), and 30‐day mortality rates (10%). Data were collected over four years, before and after the introduction of vaccines and treatment. During the early years of the pandemic, more patients were admitted to the ICU compared to later years. There was also a trend toward longer hospital stays and higher mortality rates, which seems logical during the beginning of a pandemic, but these differences were not statistically significant across the years 2020‐2024. Unvaccinated SARS‐CoV‐2 positive patients were more often admitted to the ICU and required ventilation more frequently, consistent with outcomes from other studies [13, 14], although the mortality did not differ. The outcomes of treated patients (31%) were worse than those of patients not treated with, among others, IL‐6 inhibitors, corticosteroids, and monoclonal antibodies. This suggests that the treated population was more severely ill, which is consistent with the indications for the prescribed medications. Despite vaccines and treatment, SARS‐CoV‐2 outcomes remained overall worse than influenza, with SARS‐CoV‐2 identified as an independent predictor of poor outcomes in our multivariate analysis. The greater burden of SARS‐CoV‐2 compared to seasonal influenza is consistent with other studies [15] and could partly be explained by its multisystem nature [16] and the novelty of the disease.

Although there is growing attention on the RSV vaccine, our cohort of immunocompromised patients showed limited RSV infections compared to other RVIs. RSV outcomes, including hospitalization (72%), ICU admission (5%) and 30‐day mortality (3%) were similar to those of influenza and SARS‐CoV‐2, which could justify RSV‐vaccination in SOT recipients if these outcomes were to be prevented.

Some studies suggest co‐infections do not worsen outcomes [17], while others report higher mortality in co‐infected patients compared to mono‐infected (SARS‐CoV‐2) patients in the general population [18, 19]. In our study, co‐infected patients had higher hospitalization rate compared to those with mono‐infections (84% vs. 68%, respectively, *p*  =  0.04), but no association was found between co‐infection and poor outcomes in the multivariate analysis.

The CURB‐65 score is used to predict 30‐day mortality in community‐acquired pneumonia, with higher scores indicating increased mortality risk, and has been validated for immunocompetent adults and for bacterial pneumonia [20]. Our findings suggest that CURB‐65 score can also be used to quantify severity in RVI in immunocompromised adults, as a higher CURB‐65 score was correlated with increased odds of 30‐day mortality or ICU‐admission. Similar results were reported by Müller‐Plathe et al. in kidney transplant patients with increasing mortality rates with higher CURB‐65 scores. However, the predictive accuracy was modest (area under the ROC curve [AUC] 0.698 [0.560–0.836]) [21]. Carrabba et al. also found limited prognostic value in immunocompromised patient with health‐care associated pneumonia (sensitivity of 0.731 [0.590–0.844]) [22]. Gonzalez et al. observed that a CURB‐65 score of ≥ 2 was linked to higher 28‐day mortality in cancer patients with bacterial pneumonia, but with poor discrimination between fatal and nonfatal cases [23]. Conversely, in a cohort of immunocompetent patients with SARS‐CoV‐2 pneumonia, the CURB‐65 score showed better predictive ability (AUC 0.82 [95% CI 0.73–0.91]) [24].

An average CURB‐65 score of 1.5 in our study indicated that most patients were mildly ill. Despite this, 59% of patients received antibiotics post‐PCR, suggesting overuse of antibiotics. This is a relevant finding within the context of antimicrobial stewardship, as optimal use of antibiotics is necessary to preserve their efficacy by reducing the resistance development [25]. The overuse of antibiotics was especially observed for HRV and SARS‐CoV‐2, despite a low incidence of infiltrates on chest x‐ray. However, for SARS‐CoV‐2 patients, fewer chest x‐rays were performed compared to the other RVIS. This is likely because computed tomography (CT) scan was a commonly used tool in the diagnostic workup of COVID‐19 patients [26].

No clear association was found between the number of maintenance immunosuppressive agents, recent rejection therapy or the use of calcineurin or proliferation inhibitors and the composite endpoint. Some studies suggest a possible association [5, 27], whereas others do not [28, 29]. However, reported findings should be interpreted with caution due to unknown denominators and the lack of well‐defined measures for immunosuppression intensity.

RVIs are considered as a risk factor for graft failure in SOT recipients, especially lung transplant recipients [2, 3, 7], as the infection and its compromised immune response in the transplanted organ may lead to local graft failure [30, 31]. In our study, the 30‐day rejection risk was low (4/565, 0.7%), aligning with previous studies [7]. RVI‐related rejection appears less of an issue in non‐lung transplant recipients, as severe immunosuppression alone may account for clinical outcomes in non‐lung transplant recipients.

### 4.1 Limitations

Our study has several limitations. RVI testing and the number of viruses tested per PCR were based on clinical judgement rather than a standardized protocol, potentially underrepresenting milder or less frequently tested viral infections, especially in non‐academic hospitals and before 2017 and after 2020. Data were collected retrospectively, leading to missing information, particularly regarding clinical characteristics. In addition, the exact reason for admission could not be determined. Mildly symptomatic patients who didn't seek medical attention were not included, potentially skewing the distribution of RVIs. Lastly, we lacked CT (threshold cycles) values for PCR results and data on the SARS‐CoV‐2 variant, as it was not routinely determined.

### 4.2 Strengths

Our study has several strengths. It includes a large cohort of SOT recipients from various centers and transplant types. Data on RVIs were collected over several seasons within our transplantation region, covering the full spectrum of post‐transplantation care. Including seven affiliated hospitals allowed us to capture patients seeking care elsewhere. Additionally, we included all respiratory viruses, enabling broader comparisons between groups, although not all hospitals used comprehensive assays for these viruses.

## 5 Conclusion

In conclusion, RVIs have a considerable impact on SOT recipients, with SARS‐CoV‐2 and influenza being the most common. SARS‐CoV‐2 was a major contributor to poor outcomes, while influenza showed lower, but still notable morbidity and mortality. Given the burden of these vaccine‐preventable infections, improving vaccine uptake and response is essential. The burden of RSV disease was low compared to other viral pathogens in our cohort, which may influence future vaccination strategies, particularly in immunosuppressed patients. Independent risk factors for increased morbidity and mortality included a higher CURB‐65 score, radiologic infiltrates on x‐ray and SARS‐CoV‐2 infections.

## Funding

The authors have nothing to report.

## Conflicts of Interest

The authors declare no conflicts of interest.

## Supporting information




**Supporting Figure 1**: Distribution of respiratory viruses. **Supporting Figure 2**: Number of viruses tested per PCR. **Supporting Figure 3**: Seasonality and distribution of respiratory viruses. **Supporting Figure 4**: Treatment of SARS‐CoV‐2 positive patients. **Supporting Table 1**: Co‐infections. **Supporting Table 2**: Patient characteristics of mono‐infection RVIs. **Supporting Table 3**: Number of viruses tested each year. **Supporting Table 4**: Number of viruses tested per PCR per hospital.**Supporting Table 5**: CURB‐65 score and 30‐day mortality **Supporting Table 6**: CURB‐65 score and 30‐day mortality, excluding patients with an infiltrate on chest X‐ray. **Supporting Table 7**: Outcomes in patients with a mono‐infection of SARS‐CoV‐2, compared to patients with a mono‐infection of influenza. **Supporting Table 8**: Outcomes in patients with a mono‐infection of RSV, compared to patients with a mono‐infection of influenza. **Supporting Table 9**: Outcomes in patients with a mono‐infection of RSV, compared to patients with a mono‐infection of SARS‐CoV‐2. **Supporting Table 10**: Outcomes in patients with a mono SARS‐CoV‐2 infection (n=216) and patients with a SARS‐CoV‐2 co‐infection (n=16). **Supporting Table 11**: Outcomes associated with RVI in patients with a mono‐infection influenza (n=166) and patients with a influenza co‐infection (n=19). **Supporting Table 12**: Course of disease in vaccinated and unvaccinated influenza positive patients. **Supporting Table 13**: Course of disease in vaccinated and unvaccinated SARS‐CoV‐2 patients. **Supporting Table 14**: Outcomes in treated and untreated SARS‐CoV‐2 patients. **Supporting Table 15**: Outcomes in SARS‐CoV‐2 patients by year of infection.

## Data Availability

The data that support the findings of this study are available from the corresponding author upon reasonable request.
