# Supplementary material for: Epidemiology and Clinical Impact of Confirmed Respiratory Viral Infections in Solid Organ Transplant Recipients
Source: Transpl Infect Dis. 2025 Nov 8;28(1):e70128. doi: 10.1111/tid.70128 (PMC12892827; doi:10.1111/tid.70128)
Supplement: Supplementary file 1 — Supporting Figure 1: Distribution of respiratory viruses. Supporting Figure 2: Number of viruses tested per PCR. Supporting Figure 3: Seasonality and distribution of respiratory viruses. Supporting Figure 4: Treatment of SARS‐CoV‐2 positive patients. Supporting Table 1: Co‐infections. Supporting Table 2: Patient characteristics of mono‐infection RVIs. Supporting Table 3: Number of viruses tested each year. Supporting Table 4: Number of viruses tested per PCR per hospital.Supporting Table 5: CURB‐65 score and 30‐day mortality Supporting Table 6: CURB‐65 score and 30‐day mortality, excluding patients with an infiltrate on chest X‐ray. Supporting Table 7: Outcomes in patients with a mono‐infection of SARS‐CoV‐2, compared to patients with a mono‐infection of influenza. Supporting Table 8: Outcomes in patients with a mono‐infection of RSV, compared to patients with a mono‐infection of influenza. Supporting Table 9: Outcomes in patients with a mono‐infection of RSV, compared to patients with a mono‐infection of SARS‐CoV‐2. Supporting Table 10: Outcomes in patients with a mono SARS‐CoV‐2 infection (n=216) and patients with a SARS‐CoV‐2 co‐infection (n=16). Supporting Table 11: Outcomes associated with RVI in patients with a mono‐infection influenza (n=166) and patients with a influenza co‐infection (n=19). Supporting Table 12: Course of disease in vaccinated and unvaccinated influenza positive patients. Supporting Table 13: Course of disease in vaccinated and unvaccinated SARS‐CoV‐2 patients. Supporting Table 14: Outcomes in treated and untreated SARS‐CoV‐2 patients. Supporting Table 15: Outcomes in SARS‐CoV‐2 patients by year of infection. [file TID-28-e70128-s001.pdf]

Supplements to:

Epidemiology and clinical impact of confirmed respiratory viral infections in solid organ transplant recipients

Manon L.M. Prins[1 2], Ernst D. van Dokkum[3 4], Aiko P.J. de Vries[5], Maarten E. Tushuizen[6], Danny van der Helm[5], Edwin M. Spithoven[7,8], Irene M. van der Meer[9], Eduard M Scholten[10], Albert M. Vollaard[11], Saskia le Cessie[12], Leo G. Visser[1], Geert H. Groeneveld[1 2]

1. LUCID, subdepartment of Infectious Diseases, Leiden University Medical Center, Leiden, The Netherlands
2. Department of Internal Medicine, Division Acute Internal Medicine, Leiden University Medical Center, Leiden, the Netherlands
3. Department of Public Health and Primary care, Leiden University Medical Center, Leiden, The Netherlands
4. Health Campus The Hague, Leiden University Medical Center, The Hague, The Netherlands
5. Department of Internal Medicine, Division of Nephrology and Leiden Transplant Center, Leiden University Medical Center, Leiden, the Netherlands
6. Department of Gastroenterology and Hepatology, Leiden University Medical Center, Leiden, the Netherlands
7. Department of Internal Medicine, Amphia Hospital, Breda, the Netherlands
8. Current affiliation: Department of Internal Medicine, Rijnstate Hospital, Arnhem, the Netherlands
9. Department of Nephrology, Haga Teaching Hospital, the Hague, the Netherlands
10. Department of Nephrology, Haaglanden Medical Center, the Hague, the Netherlands
11. Centre for Infectious Disease Control, National Institute for Public Health and the Environment, Bilthoven, The Netherlands.
12. Department of Clinical Epidemiology, Leiden University Medical Center, Leiden, the Netherlands

Table of Contents

Supplemental Figure 1. Distribution of respiratory viruses. 4

Supplemental Figure 2. Number of viruses tested per PCR 5

Supplemental Figure 3. Seasonality and distribution of respiratory viruses 6

Supplemental Figure 4. Treatment of SARS-CoV-2 positive patients 7

Supplemental Table 1. Co-infections 8

Supplemental Table 2. Patient characteristics of mono-infection RVIs 9

Supplemental Table 3. Number of viruses tested each year 12

Supplemental Table 4. Number of viruses tested per PCR per hospital 13

Supplemental Table 5. CURB-65 score and 30-day mortality 14

Supplemental table 6. CURB-65 score and 30-day mortality, excluding patients with an infiltrate on chest X-ray 14

Supplemental Table 7. Outcomes in patients with a mono-infection of SARS-CoV-2, compared to patients with a mono-infection of influenza 15

Supplemental Table 8. Outcomes in patients with a mono-infection of RSV, compared to patients with a mono-infection of influenza 15

Supplemental Table 9. Outcomes in patients with a mono-infection of RSV, compared to patients with a mono-infection of SARS-CoV-2 16

Supplemental Table 10. Outcomes in patients with a mono SARS-CoV-2 infection (n=216) and patients with a SARS-CoV-2 co-infection (n=16) 16

Supplemental Table 11. Outcomes associated with RVI in patients with a mono-infection influenza (n=166) and patients with a influenza co-infection (n=19) 17

Supplemental Table 12. Course of disease in vaccinated and unvaccinated influenza positive patients 18

Supplemental Table 13. Course of disease in vaccinated and unvaccinated SARS-CoV-2 patients **18**

Supplemental Table 14. Outcomes in treated and untreated SARS-CoV-2 patients **19**

Supplemental Table 15. Outcomes in SARS-CoV-2 patients by year of infection **19**

Supplemental Figure 1. Distribution of respiratory viruses.

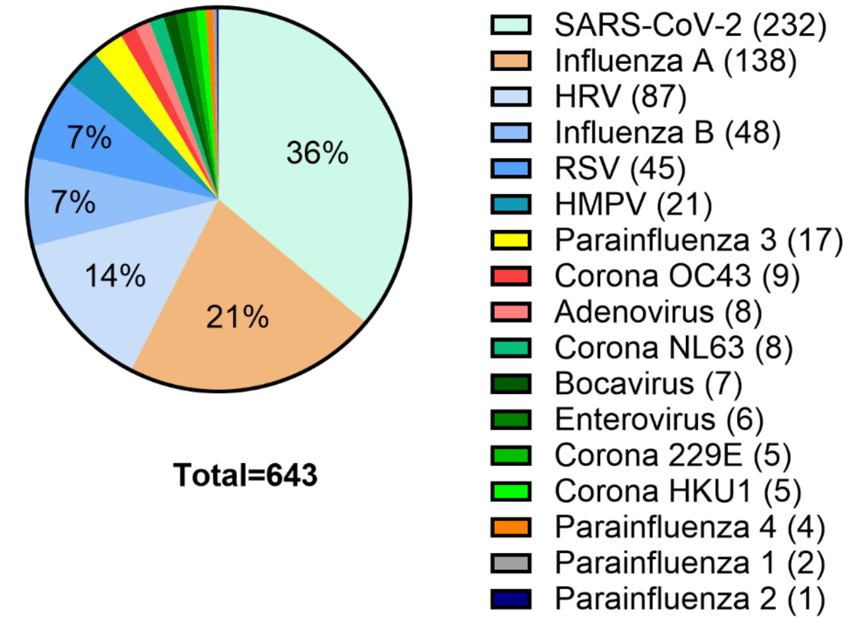

Distribution of the different viruses (n=643) in solid organ transplant recipients in percentages among 603 virus-positive specimens collected from 2013-2024. The number in parentheses represents the count of cases. Thirty-eight patients had a co-infection. Of these, thirty-six had an infection with two pathogens and two had an infection with three pathogens.

Abbreviations: RSV, respiratory syncytial virus; SARS-CoV-2, severe acute respiratory syndrome coronavirus-2; HRV, human rhinovirus; HMPV, human metapneumovirus

Supplemental Figure 2. Number of viruses tested per PCR

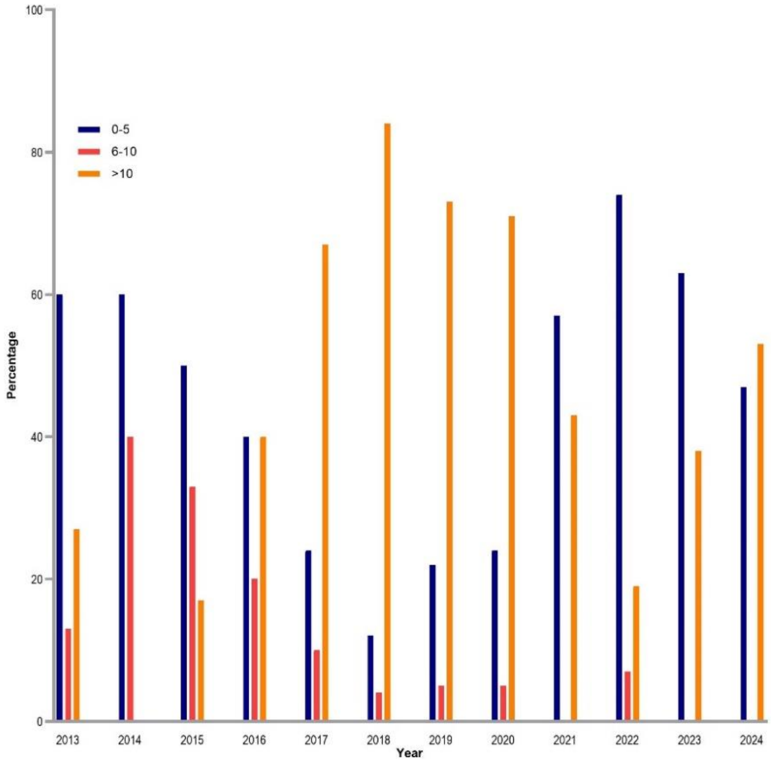

Number of viruses tested per PCR test during the years 2013-2024, divided into three categories: 0 to 6 viruses per PCR, 6 to 11 viruses per PCR and 11 or more viruses per PCR. Percentages are shown.

Supplemental Figure 3. Seasonality and distribution of respiratory viruses

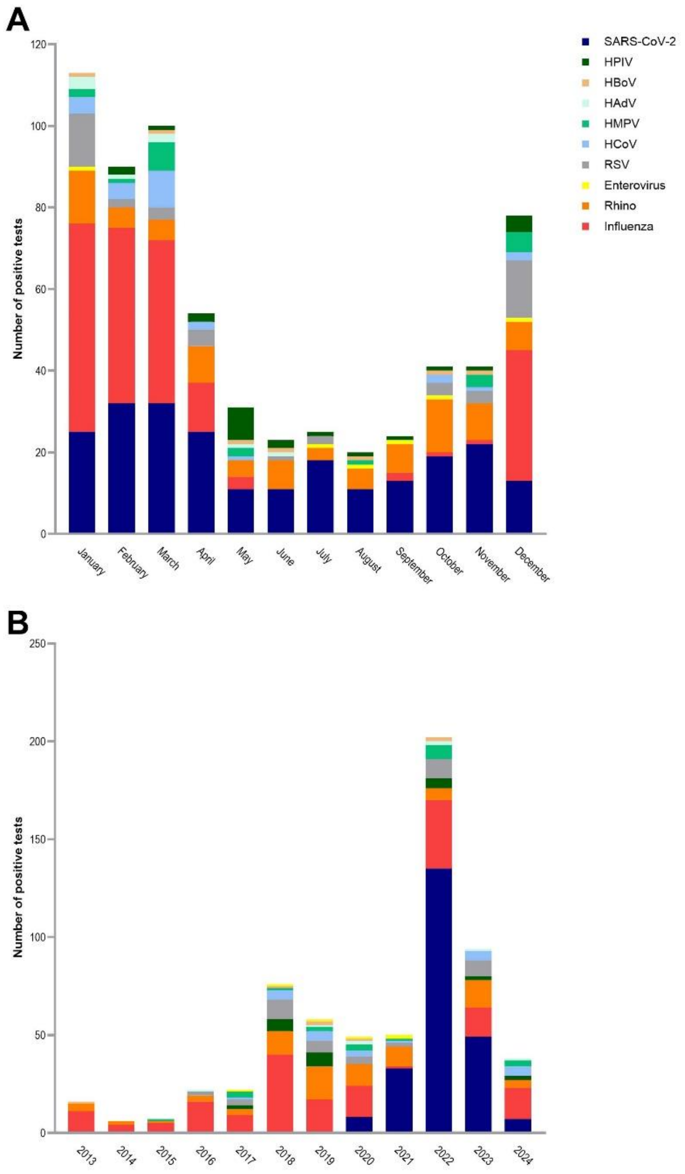

Seasonal patterns of RVIs: overall number, type of positive viral test and distribution of respiratory viruses in solid organ transplant recipients among 603 virus-positive specimens collected from 2013-2024. (A) Monthly rate of respiratory virus positivity. (B) Yearly rate of respiratory virus positivity.
Abbreviations: SARS-CoV-2, severe acute respiratory syndrome coronavirus-2; HPIV, human parainfluenza virus; HBoV, human bocavirus; HAdV, human adenovirus; HMPV, human metapneumovirus; HCoV, human coronavirus; RSV, respiratory syncytial virus; HRV, human rhinovirus

Supplemental Figure 4. Treatment of SARS-CoV-2 positive patients

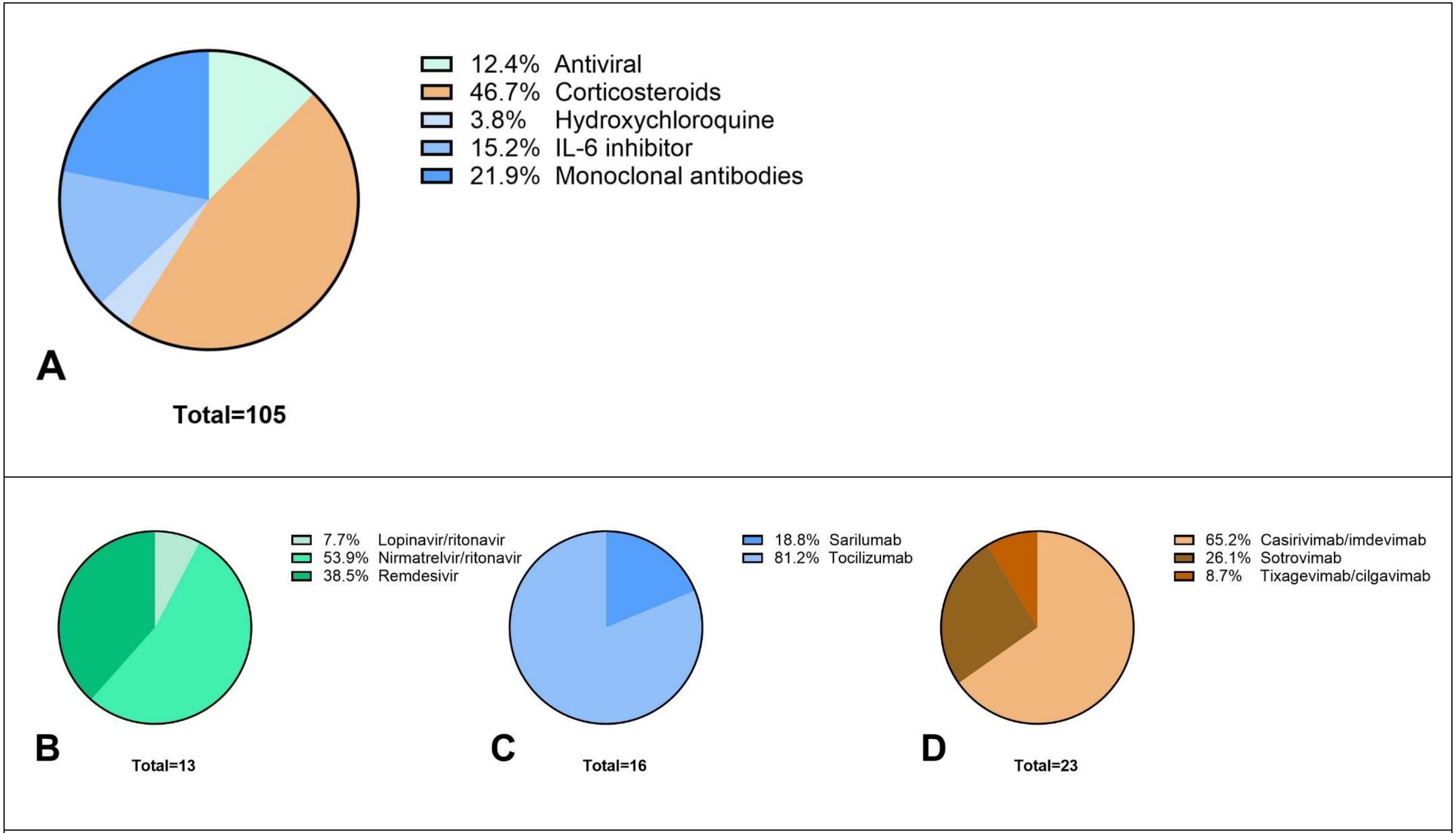

A. Total number of medication prescriptions (n=105) among 71 of the 232 SARS-CoV-2 positive patients.

B. Number of prescribed antivirals<br>C. Number of prescribed IL-6 inhibitors<br>D. Number of prescribed monoclonal antibodies

Supplemental Table 1. Co-infections

n	Virus 1	Virus 2	Virus 3
1	Influenza A	Influenza B	
6	Influenza A	SARS-CoV-2	
3	Influenza A	HRV	
1	Influenza A	HCoV OC43	
1	Influenza A	HMPV	
1	Influenza A	HCoV HKU1	HCoV NL63
1	Influenza B	HMPV	
1	Influenza B	HCoV HKU1	
1	Influenza B	HRV	
1	Influenza B	HPIV	
1	Influenza B	Enterovirus	
1	Influenza B	SARS-CoV-2	HBoV
6	SARS-CoV-2	RSV	
1	SARS-CoV-2	HRV	
1	SARS-CoV-2	HPIV	
1	SARS-CoV-2	HBoV	
3	HRV	enterovirus	
2	HRV	HAdV	
1	HRV	HBoV	
1	HRV	HCoV NL63	
1	HCoV 229E	HMPV	
1	HCoV NL63	HCoV HKU1	
1	HCoV NL63	HAdV	

Number of co-infections.
Abbreviations: SARS-CoV-2, severe acute respiratory syndrome coronavirus-2; HPIV, human parainfluenza virus; HBoV, human bocavirus; HAdV, human adenovirus; HMPV, human metapneumovirus; HCoV, human coronavirus; RSV, respiratory syncytial virus; HRV, human rhinovirus; influenza, influenza virus

Supplemental Table 2. Patient characteristics of mono-infection RVIs

	Total (n=565)[~]	SARS-CoV-2 (n=216)	Influenza (n=166)	HRV (n=75)	RSV (n=39)	HPIV (n=22)	HCoV (n=18)	HMPV (n=18)	Residual[b] (n=11)	p
Age, mean (SD)	59 (14)	60 (14)	56 (14)	60 (13)	63 (12)	58 (17)	58 (12)	63 (14)	56 (16)	0.04
Male sex, n (%)	331 (59)	132 (61)	93 (56)	45 (60)	22 (56)	10 (46)	8 (44)	13 (72)	8 (73)	0.48
BMI, mean (SD)	25.6 (4.9)	25.5 (4.5)	26.2 (5.7)	24.9 (4.8)	25.6 (4.5)	24.7 (5.0)	25.6 (5.2)	25.4 (5.0)	23.5 (4.3)	0.50
Time from transplantation in years, median (IQR)	5 (2-11)	5 (2-11)	6 (2-12)	4 (1-9)	6 (3-10)	4 (1-10)	4 (1-7)	10.5 (5-21)	9 (0-15)	0.03
Co-morbidities, n (%)	503 (89.0)	190 (88.0)	147 (88.6)	70 (93.3)	35 (89.7)	19 (86.4)	16 (88.9)	17 (94.4)	9 (81.8)	0.88
Pre-existent CVD disease	469 (93.2)	178 (93.7)	138 (93.9)	65 (92.9)	32 (91.4)	17 (89.5)	15 (93.8)	15 (88.2)	9 (100)	0.99
Pre-existent lung disease	155 (30.8)	52 (27.4)	37 (25.2)	26 (37.1)	14 (40.0)	10 (52.6)	6 (37.5)	7 (41.2)	3 (33.3)	0.10
Pre-existent DM	230 (45.7)	97 (51.1)	62 (42.2)	30 (42.9)	16 (45.7)	8 (42.1)	9 (56.3)	5 (29.4)	3 (33.3)	0.63
Type transplantation, n (%)										0.75
Kidney	470 (83.2)	185 (85.6)	134 (80.7)	62 (82.7)	35 (89.7)	16 (72.7)	16 (88.9)	14 (77.8)	8 (72.7)	
Liver	71 (12.6)	20 (9.3)	26 (15.7)	11 (14.7)	3 (7.7)	4 (18.2)	2 (11.1)	2 (11.1)	3 (27.3)	
Combined[P]	20 (3.5)	9 (4.2)	5 (3.0)	1 (1.1)	1 (2.6)	2 (9.1)	-	2 (11.1)	-	
Pancreas or islets of Langerhans[#]	4 (0.7)	2 (0.9)	1 (0.6)	1 (1.1)	-	-	-	-	-	
Induction immunosuppression, n (%)*										0.50
IL-2 inhibitor	357 (88.8)	147 (89.6)	101 (88.6)	49 (90.7)	24 (85.7)	13 (86.7)	12 (85.7)	7 (100)	4 (66.7)	
ATG	1 (0.2)	1 (0.6)	-	-	-	-	-	-	-	
Alemtuzumab	38 (9.5)	15 (9.1)	9 (7.9)	5 (9.3)	4 (14.3)	2 (13.3)	2 (14.3)	-	1 (16.7)	
Other	6 (1.5)	1 (0.6)	4 (3.5)	-	-	-		-	1 (16.7)	
No. of immunosuppressive agents										0.15
1	54 (9.6)	20 (9.3)	17 (10.2)	5 (6.7)	6 (15.4)	1 (4.5)	1 (5.6)	3 (16.7)	1 (9.1)	
2	277 (49.0)	101 (46.8)	78 (47.0)	36 (48.0)	25 (64.1)	9 (40.9)	8 (44.4)	13 (72.2)	17 (63.6)	
3	234 (41.4)	95 (44.0)	71 (42.8)	34 (45.3)	8 (20.5)	12 (54.5)	9 (50.0)	2 (11.1)	3 (27.3)	
Maintenance immunosuppressive agents										
Corticosteroids	495 (87.6)	188 (87.0)	146 (88.0)	67 (89.3)	33 (84.6)	22 (100)	14 (77.8)	17 (94.4)	8 (72.7)	0.30
Calcineurin inhibitors	425 (75.2)	165 (76.4)	130 (78.3)	55 (73.3)	26 (66.7)	15 (68.2)	16 (88.9)	9 (50.0)	9 (81.8)	0.12
Proliferation inhibitors	346 (61.2)	140 (64.8)	97 (58.4)	50 (66.7)	16 (41.0)	15 (68.2)	12 (66.7)	9 (50.0)	7 (63.6)	0.14
MTOR inhibitors	38 (6.7)	13 (6.0)	10 (6.0)	7 (9.3)	4 (10.3)	2 (9.1)	2 (11.1)	-	-	0.69
Previous rejection therapy										0.02
<6 months ago	12 (2.1)	2 (0.9)	2 (1.2)	3 (4.0)	1 (2.6)	2 (9.1)	2 (11.1)	-	-	
Once	104 (18.4)	31 (14.4)	41 (24.7)	15 (20.0)	3 (7.7)	4 (18.2)	3 (16.7)	4 (22.2)	3 (27.3)	
Never	449 (79.5)	183 (84.7)	123 (74.1)	57 (76.0)	35 (89.7)	16 (72.7)	13 (72.2)	14 (77.8)	8 (72.7)	
Type of rejection therapy*	N=559									
Solumedrol	96 (17.2)	25 (11.7)	36 (21.8)	14 (19.2)	4 (10.3)	6 (27.3)	4 (22.2)	4 (22.2)	3 (27.3)	0.12
Alemtuzumab	28 (5.0)	5 (2.3)	11 (6.7)	5 (6.8)	2 (5.1)	-	1 (5.6)	2 (11.1)	2 (18.2)	0.13

ATG	20 (3.6)	3 (1.4)	11 (6.7)	3 (4.1)	-	1 (4.5)	-	1 (5.6)	1 (9.1)	0.14
Other[&]	29 (5.2)	6 (2.8)	11 (6.7)	3 (4.1)	2 (5.1)	1 (4.5)	3 (16.7)	1 (5.6)	2 (18.2)	0.10
Time between rejection therapy and PCR in years, median (IQR)	4 (1-13.8)	6 (3.0-13.0)	7 (3-19)	2.5 (0.0-5.0)	4 (2-6)	2 (0-7)	1 (0-3)	2 (0.5-17.5)	8 (3-10)	0.10
Highly immunosuppressed[a]	235 (41.6)	95 (44.0)	72 (43.4)	34 (45.3)	8 (20.5)	12 (54.5)	9 (50.0)	2 (11.1)	3 (27.3)	0.01
Clinical presentation, n (%)										
Fever	326 (57.7)	117 (54.2)	120 (72.3)	36 (48.0)	23 (59.0)	7 (31.8)	7 (38.9)	11 (61.1)	5 (45.5)	0.009
Coughing	397 (70.3)	116 (53.7)	140 (84.3)	55 (73.3)	35 (89.7)	18 (81.8)	12 (66.7)	16 (88.9)	5 (45.5)	<0.001
Sore throat	78 (13.8)	20 (9.3)	35 (21.1)	12 (16.0)	3 (7.7)	3 (13.6)	2 (11.1)	1 (5.6)	2 (18.2)	0.35
Dyspnoea	221 (39.1)	77 (35.6)	55 (33.1)	39 (52.0)	22 (56.4)	9 (40.9)	7 (38.9)	10 (55.6)	2 (18.2)	0.15
Common cold	152 (26.9)	35 (16.2)	62 (37.3)	21 (28.0)	15 (38.5)	5 (22.7)	8 (44.4)	4 (22.2)	2 (18.2)	0.009
Myalgia	101 (17.9)	30 (13.9)	48 (28.9)	4 (5.3)	6 (15.4)	5 (22.7)	2 (11.1)	3 (16.7)	3 (27.3)	0.02
Headache	98 (17.3)	41 (19.0)	31 (18.7)	7 (9.3)	6 (15.4)	3 (13.6)	6 (33.3)	2 (11.1)	2 (18.2)	0.79
General malaise	164 (29.0)	58 (31.5)	43 (25.9)	19 (25.3)	6 (15.4)	10 (45.5)	9 (50.0)	7 (38.9)	2 (18.2)	0.06
Other^	55 (9.7)	25 (11.6)	14 (8.4)	9 (12.0)	3 (7.7)	-	1 (5.6)	3 (16.7)	-	0.46
Time between first symptoms and PCR in days, median (IQR)	3 (1-7)	3 (1-10)	2 (1-6)	6 (2-11)	4 (2-7)	3 (2-7)	3 (2-5)	4 (3-5)	4 (2-13)	0.008
Time between first symptoms and admission in hospital in days, mean (SD)	4.8 (7.7)	6.0 (9.0)	3.2 (6.2)	5.5 (6.7)	5.3 (9.0)	3.9 (3.1)	0.9 (7.9)	3.5 (2.3)	4.8 (6.7)	0.11
CURB-65 score, mean (SD)	1.5 (0.9)	1.5 (1.0)	1.3 (0.9)	1.5 (0.9)	1.7 (0.9)	1.5 (0.9)	1.5 (0.9)	1.8 (1.0)	1.3 (0.9)	0.30
Radiology, n (%)	421 (74.5)	136 (63.0)	133 (80.1)	58 (77.3)	35 (89.7)	19 (86.4)	14 (77.8)	16 (88.9)	10 (90.9)	<0.001
Infiltrate	130 (30.9)	50 (36.8)	29 (21.8)	21 (36.2)	7 (20.0)	4 (21.1)	5 (35.7)	7 (43.8)	7 (70.0)	0.007
Treated with oseltamivir	-	-	110 (66.3)	-	-	-	-	-	-	
Antibiotics prior to admission, n (%)	171 (30.3)	63 (29.2)	35 (21.1)	36 (48.0)	11 (28.2)	10 (45.5)	5 (27.8)	6 (33.3)	5 (45.5)	0.003
Antibiotics after admission, n (%)	334 (59.1)	109 (50.5)	90 (54.2)	59 (78.7)	28 (71.8)	15 (68.2)	11 (61.1)	13 (72.2)	9 (81.8)	<0.001

Univariate analysis was performed for the baseline characteristics using either the ANOVA test, Kruskal-Wallis test or Chi-squared test. The p-values shown in this table have not yet been Bonferroni corrected . Post-hoc analysis was performed in case of significance differences found by univariate analysis (bold variables). These values are not shown.

BMI, body mass index; CVD, cardiovascular disease; DM, diabetes mellitus; IL-, interleukin-2; ATG, anti-thymocyte globulin; MTOR, mammalian target of rapamycin; SARS-CoV-2, severe acute respiratory syndrome coronavirus-2; HPIV, human parainfluenza virus; HMPV, human metapneumovirus; HCoV, human coronavirus; RSV, respiratory syncytial virus; HRV, human rhinovirus; influenza, influenza virus

[~] Only patients with a mono-infections are included

[P]Combined transplantation include kidney-pancreas (n=12) and kidney-liver (n=11)

[#]Pancreas transplantation: n=2; Islets of Langerhans: n=2

*valid percentages, as numbers do not always add up to 603 as there are some missing data.

[&]Other types of rejection therapy included muromonab-CD3 (OKT3), plasmapheresis, IVIG, rituximab, addition of a third agent, switch to tacrolimus

[a] Included patients who used lymphocyte depleting agents less than six months ago (n=5) and/or patients who used three immunosuppressive agents (n=247). Four of the five patients who used lymphocyte depleting agents used three immunosuppressive agents as well.

^ Other symptoms include tiredness, chest pain, cold chills, ear pain, delirium, need of oxygen

[b] Residual group consists of HAdV (n=5), HBoV (n=4), enterovirus (n=2)

Supplemental Table 3. Number of viruses tested each year

Year	Number of viruses tested per PCR													Total number of PCR tests
	1	2	3	4	5	6	9	10	11	13	15	16	17	
2013	2 (13.3)	5 (33.3)	2 (13.3)	0	0	0	2 (13.3)	0	0	1 (6.7)	3 (20.0)	0	0	15
2014	0	0	2 (40.0)	0	1 (20.0)	0	2 (40.0)	0	0	0	0	0	0	5
2015	0	3 (50.0)	0	0	0	0	1 (16.7)	1 (16.7)	0	0	1 (16.7)	0	0	8
2016	0	6 (30.0)	2 (10.0)	0	0	0	2 (10.0)	2 (10.0)	3 (15.0)	0	4 (20.0)	1 (5.0)	0	20
2017	0	5 (23.8)	0	0	0	0	2 (9.5)	0	0	0	14 (66.7)	0	0	21
2018	0	2 (2.7)	7 (9.6)	0	0	0	2 (2.7)	1 (1.4)	0	0	57 (78.1)	4 (5.5)	0	73
2019	0	3 (5.5)	8 (14.5)	1 (1.8)	0	0	3 (5.5)	0	0	0	39 (70.9)	1 (1.8)	0	55
2020	0	5 (12.2)	3 (7.3)	2 (4.9)	0	2 (4.9)	0	0	0	1 (2.4)	15 (36.6)	10 (24.4)	3 (7.3)	41
2021	0	0	1 (2.0)	26 (53.1)	1 (2.0)	0	0	0	0	0	1 (2.0)	17 (34.7)	3	49
2022	0	1 (0.5)	3 (1.5)	137 (70.6)	3 (1.5)	0	0	14 (7.2)	4 (2.1)	0	2 (1.0)	25 (12.9)	5 (2.6)	194
2023	0	0	0	54 (61.4)	1 (1.1)	0	0	0	9 (10.2)	0	2 (2.3)	16 (18.2)	6 (6.8)	88
2024	0	0	0	17 (47.2)	0	0	0	0	0	0	0	13 (36.1)	6 (16.7)	36
Total	2 (0.3)	30 (5.0)	28 (4.6)	237 (39.3)	6 (1.0)	2 (0.3)	14 (2.3)	18 (3.0)	16 (2.7)	2 (0.3)	138 (22.9)	87 (14.4)	23 (3.8)	603

Overview of the total number of viruses tested per PCR each year, aggregated across all hospitals. N and corresponding percentages are presented.

Supplemental Table 4. Number of viruses tested per PCR per hospital

	Mean (IQR)	n
Hospital 1	4 (3-4)	16
Hospital 2	10 (9-11)	50
Hospital 3	15 (4-15)	16
Hospital 4	4 (3-4)	80
Hospital 5	4 (3-4)	22
Hospital 6	15 (4-15)	348
Hospital 7	7 (4-15)	24
Hospital 8	15 (4-16)	47

Supplemental Table 5. CURB-65 score and 30-day mortality

CURB-65 score	30-day mortality
0	2/56 (3.6%)
1	4/216 (1.9%)
2	12/138 (8.7%)
3	8/57 (14.0%)
4	3/8 (37.5%)
5	0/1 (0%)

Relationship between CURB-65 score and 30-day mortality in 460 patients with 603 respiratory viral infections.
Abbreviations: CURB-65 severity score: C, new-onset confusion; U, urea>7mmol/L; R, respiratory rate ≥30/min, B, blood pressure (systolic < 90 mmHg or diastolic ≤60 mmHg); 65, age ≥65 years.

Supplemental table 6. CURB-65 score and 30-day mortality, excluding patients with an infiltrate on chest X-ray

CURB-65 score	30-day mortality
0	1/32 (3.1%)
1	2/110 (1.8%)
2	4/78 (5.1%)
3	3/31 (9.7%)
4	1/3 (33.3%)
5	0/1 (0%)

Relationship between CURB-65 score and 30-day mortality in 255 patients with respiratory viral infections without an infiltrate on chest X-ray.
Abbreviations: CURB-65 severity score: C, new-onset confusion; U, urea>7mmol/L; R, respiratory rate ≥30/min, B, blood pressure (systolic < 90 mmHg or diastolic ≤60 mmHg); 65, age ≥65 years.

Supplemental Table 7. Outcomes in patients with a mono-infection of SARS-CoV-2, compared to patients with a mono-infection of influenza

Outcome variable	Total (n=382)	SARS-CoV-2 (n=216)	Influenza (n=166)	OR (95% CI)	p[a]
Hospital admission	256 (67.0)	154 (71.3)	102 (61.4)	1.56 (1.01-2.40)	0.04
Length of stay in days, median (IQR)	3 (2-8)	4 (2-12)	3 (2-5)	-	<0.001
Admission to ICU	30 (7.9)	24 (11.1)	6 (3.6)	3.33 (1.33-8.36)	0.007
Mechanical ventilation	27 (7.1)	23 (10.6)	4 (2.4)	4.83 (1.64-14.24)	0.002
30-day mortality	25 (6.6)	22 (10.2)	3 (1.8)	6.19 (1.82-21.07)	<0.001
Composite endpoint	39 (10.2)	33 (15.3)	6 (3.6)	4.81 (1.96-11.77)	<0.001
30-day rejection	2 (0.5)	0 (0)	2 (1.2)	0.99 (0.97-1.01)	0.19

Counts and percentages are presented, unless otherwise stated.

[a] Mann-Whitney U test or Chi-squared test or Fisher's exact test in the cells with a value of <6

The composite endpoint consists of admission to ICU and 30-day mortality.

Abbreviations: ICU, intensive care unit; IQR, interquartile range; OR, odds ratio

Supplemental Table 8. Outcomes in patients with a mono-infection of RSV, compared to patients with a mono-infection of influenza

Outcome variable	Total (n=205)	RSV (n=39)	Influenza (n=166)	OR (95% CI)	p[a]
Hospital admission	130 (63.4)	28 (71.8)	102 (61.4)	1.67 (0.77-3.60)	0.19
Length of stay in days, median (IQR)	3 (2-6)	5 (2-8)	3 (2-5)	-	0.11
Admission to ICU*	8 (3.9)	2 (5.1)	6 (3.6)	1.61 (0.30-8.63)	0.63
Mechanical ventilation	6 (2.9)	2 (5.1)	4 (2.4)	2.72 (0.44-16.88)	0.27
30-day mortality*	4 (2.0)	1 (2.6)	3 (1.8)	2.00 (0.18-22.64)	0.57
Composite endpoint	8 (3.9)	2 (5.1)	6 (3.6)	1.61 (0.30-8.63)	0.63
30-day rejection	2 (1)	0 (0)	2 (1.2)	0.99 (0.97-1.01)	0.47

Counts and percentages are presented, unless otherwise stated.

*valid percentages, as there are some missing data.

[a] Mann-Whitney U test or Chi-squared test or Fisher's exact test in the cells with a value of <6

The composite endpoint consists of admission to ICU and 30-day mortality.

Abbreviations: ICU, intensive care unit; IQR, interquartile range; OR, odds ratio

Supplemental Table 9. Outcomes in patients with a mono-infection of RSV, compared to patients with a mono-infection of SARS-CoV-2

Outcome variable	Total (n=255)	RSV (n=39)	SARS-CoV-2 (n=216)	OR (95% CI)	p[a]
Hospital admission	182 (71.4)	28 (71.8)	154 (71.3)	1.03 (0.48-2.19)	0.95
Length of stay in days, median (IQR)	4.5 (2-11)	5 (2-8)	4 (2-12)	-	0.77
Admission to ICU*	26 (10.2)	2 (5.1)	24 (11.1)	0.43 (0.10-1.91)	0.39
Mechanical ventilation	25 (9.8)	2 (5.1)	23 (10.6)	0.45 (0.10-2.01)	0.39
30-day mortality*	23 (9.1)	1 (2.6)	22 (10.2)	0.23 (0.03-1.77)	0.22
Composite endpoint	35 (13.7)	2 (5.1)	33 (15.3)	0.30 (0.07-1.30)	0.09
30-day rejection	0 (0)	0 (0)	0 (0)	-	-

Counts and percentages are presented, unless otherwise stated.
*valid percentages, as there are some missing data.
[a] Mann-Whitney U test or Chi-squared test or Fisher's exact test in the cells with a value of <6
The composite endpoint consists of admission to ICU and 30-day mortality.
ICU, intensive care unit; IQR, interquartile range; OR, odds ratio

Supplemental Table 10. Outcomes in patients with a mono SARS-CoV-2 infection (n=216) and patients with a SARS-CoV-2 co-infection (n=16)

Outcome variable	Mono-infection (n=216)	Co- infection (n=16)	OR (95% CI)	p[a]
Hospital admission	154 (71.3)	12 (75.0)	1.21 (0.38-3.89)	0.75
Length of stay in days, median (IQR)	4 (2-12)	7 (3-13)	-	0.97
Admission to ICU	24 (11.1)	3 (18.8)	1.85 (0.49-6.95)	0.36
Mechanical ventilation	23 (10.6)	3 (18.8)	1.94 (0.51-7.31)	0.32
30-day mortality	22 (10.2)	3 (18.8)	2.02 (0.54-7.66)	0.29
Composite endpoint	33 (15.3)	5 (31.3)	2.52 (0.82-7.73)	0.10
30-day rejection	0 (0)	0 (0)	-	-

Counts and percentages are presented, unless otherwise stated.
[a] Mann-Whitney U test or Chi-squared test or Fisher's exact test in the cells with a value of <6
The composite endpoint consists of admission to ICU and 30-day mortality.
Abbreviations: ICU, intensive care unit; IQR, interquartile range; OR, odds ratio

Supplemental Table 11. Outcomes associated with RVI in patients with a mono-infection influenza (n=166) and patients with a influenza co-infection (n=19)

Outcome variable	Mono-infection (n=166)	Co- infection (n=19)	OR (95% CI)	p[a]
Hospital admission	102 (61.4)	18 (94.7)	11.3 (1.47-86.7)	0.004
Length of stay in days, median (IQR)	3 (2-5)	3 (2-10.8)	-	0.36
Admission to ICU	6 (3.6)	1 (5.3)	1.48 (0.17-13.01)	0.72
Mechanical ventilation	4 (2.4)	1 (5.3)	2.25 (0.24-21.24)	0.47
30-day mortality	3 (1.8)	1 (5.3)	3.02 (0.30-30.56)	0.33
Composite endpoint	6 (3.6)	2 (10.5)	3.14 (0.59-16.78)	0.16
30-day rejection	2 (1.2)	0 (0)	0.99 (0.97-1.01)	0.63

Counts and percentages are presented, unless otherwise stated.

[a] Mann-Whitney U test or Chi-squared test or Fisher's exact test (in the cells with a value of <6)

The composite endpoint consists of admission to ICU and 30-day mortality.

Abbreviations: ICU, intensive care unit; IQR, interquartile range; composite endpoint, 30-day mortality and/or ICU admission

Supplemental Table 12. Course of disease in vaccinated and unvaccinated influenza positive patients

	Overall (n=177)	Vaccinated (n=75)	Unvaccinated (n=102)	p[a]
Hospital admission	113 (63.8)	52 (69.3)	61 (59.8)	0.19
Length of stay in days, median (IQR)	3 (2-5)	3 (2-7)	4 (2-11)	0.19
Admission to ICU*	6 (3.4)	2 (2.7)	4 (3.9)	0.65
Mechanical ventilation	5 (2.8)	2 (2.7)	3 (2.9)	0.91
30-day mortality	3 (1.7)	1 (1.3)	2 (2.0)	0.75
Composite endpoint	6 (3.4)	2 (2.7)	4 (3.9)	0.65
30-day rejection[&]	2 (1.1)	0 (0)	2 (2.0)	0.51

Counts and percentages are presented, unless otherwise stated.
*Valid percentages are presented as there is missing data
[a]Chi-squared test, Fisher's exact test or Mann-Whitney U test.
The composite endpoint consists of admission to ICU and 30-day mortality.
ICU, intensive care unit; IQR, interquartile range; OR, odds ratio

Supplemental Table 13. Course of disease in vaccinated and unvaccinated SARS-CoV-2 patients

	Overall (n=232)	Vaccinated (n=177)	Unvaccinated (n=26)	p[a]
Hospital admission	166 (71.6)	126 (71.2)	19 (73.1)	0.84
Length of stay in days, median (IQR)	4 (2-13)	4 (2-11)	5 (2-17)	0.31
Admission to ICU	27 (11.6)	14 (7.9)	6 (23.1)	0.02
Mechanical ventilation	26 (11.2)	14 (7.9)	6 (23.1)	0.02
30-day mortality	25 (10.8)	17 (9.7)	3 (11.5)	0.77
Composite endpoint	38 (16.4)	24 (13.6)	6 (23.1)	0.20
30-day rejection[&]	0 (0)	0 (0)	0 (0)	-

In total, 232 patients tested positive for SARS-CoV-2. Of these, 177 were vaccinated and 26 were unvaccinated. The vaccination status of 29 patients was unknown.
Counts and percentages are presented, unless otherwise stated.
[a]Chi-squared test, Fisher's exact test or Mann-Whitney U test.
The composite endpoint consists of admission to ICU and 30-day mortality.
ICU, intensive care unit; IQR, interquartile range; OR, odds ratio

Supplemental Table 14. Outcomes in treated and untreated SARS-CoV-2 patients

Outcome variable	Total (n=232)	Treated (n=71)	Untreated (n=160)	p[a]
Hospital admission	166 (71.6)	65 (93.0)	99 (61.9)	<0.001
Length of stay in days, median (IQR)	4 (2-13)	8.5 (2.8-25.3)	3.0 (2.0-7.0)	<0.001
Admission to ICU	27 (11.6)	20 (28.2)	6 (3.8)	<0.001
Mechanical ventilation	26 (11.2)	19 (26.8)	6 (3.8)	<0.001
30-day mortality*	25 (10.8)	16 (22.9)	8 (5.0)	<0.001
Composite endpoint	38 (16.4)	25 (35.2)	12 (7.5)	<0.001
30-day rejection	0 (0)	0 (0)	0 (0)	-

Counts and percentages are presented, unless otherwise stated.
*valid percentages, as there are some missing data.
[a] Mann-Whitney U test or Chi-squared test or Fisher's exact test in the cells with a value of <6
The composite endpoint consists of admission to ICU and 30-day mortality.
ICU, intensive care unit; IQR, interquartile range; OR, odds ratio

Supplemental Table 15. Outcomes in SARS-CoV-2 patients by year of infection

Outcome variable	2020 (n=8)	2021 (n=33)	2022 (n=135)	2023 (n=49)	2024 (n=7)	p[a]
Hospital admission	7 (87.5)	26 (78.8)	89 (65.9)	37 (75.5)	7 (100%)	0.13
Length of stay in days, median (IQR)	11 (2-17)	7 (3.8-22.3)	3 (2-11)	5 (2-17.5)	2 (1-4)	0.13
Admission to ICU	3 (37.5)	7 (21.2)	11 (8.1)	5 (10.2)	1 (14.3)	0.04
Mechanical ventilation	3 (37.5)	6 (18.2)	11 (8.1)	5 (10.2)	1 (14.3)	0.07
30-day mortality*	2 (25.0)	4 (12.1)	14 (10.4)	5 (10.2)	0	0.63
Composite endpoint	4 (50)	7 (21.2)	19 (14.1)	7 (14.3)	1 (14.3)	0.10
30-day rejection	0 (0)	0 (0)	0 (0)	0 (0)	0 (0)	-

Counts and percentages are presented, unless otherwise stated.
*valid percentages, as there are some missing data.
[a] Kruskal- Wallis test or Chi-squared test
The composite endpoint consists of admission to ICU and 30-day mortality.
ICU, intensive care unit; IQR, interquartile range; OR, odds ratio
